# Supplementary material for: Deciphering ion transport and ATPase coupling in the intersubunit tunnel of KdpFABC
Source: Nat Commun. 2021 Aug 24;12:5098. doi: 10.1038/s41467-021-25242-x (PMC8385062; doi:10.1038/s41467-021-25242-x)
Supplement: Supplementary file 4 — Description of additional supplementary files [file 41467_2021_25242_MOESM4_ESM.docx]

Description of additional supplementary files

Title: Supplementary Movie 1

Description: Progression of K+ through the intersubunit tunnel of KdpFABC in atomistic MD simulations. Ion and protein coordinates from 7NNL [https://www.rcsb.org/structure/7NNL] were equilibrated, and then the four structural K+ closest to the KdpB CBS were removed. During unrestrained MD, two of the remaining ions show a rapid progression through the intersubunit tunnel towards the KdpB CBS, with a first ion passing KdpBF232 and entering the PBS, while the second ion stalls before the constriction in the DBS.
